# Prognostic nutritional index and prognosis of patients with coronary artery disease: A systematic review and meta-analysis

**DOI:** 10.3389/fnut.2023.1114053

**Published:** 2023-03-16

**Authors:** Shengjing Zhang, Huanfen Wang, Saiya Chen, Shengsheng Cai, Shigeng Zhou, Congling Wang, Xiuyuan Ni

**Affiliations:** ^1^Department of Geriatrics, Affiliated Wenzhou Hospital of Traditional Chinese Medicine, Zhejiang Chinese Medical University, Wenzhou, China; ^2^Department of Geriatrics, Wenzhou Geriatric Hospital, Wenzhou, China

**Keywords:** acute coronary syndrome, myocardial infarction, PCI, nutrition, prognosis, mortality

## Abstract

**Background:**

This review assessed if prognostic nutritional index (PNI) can predict mortality and major adverse cardiac events (MACE) in coronary artery disease (CAD) patients.

**Methods:**

PubMed, Web of Science, Scopus, and Embase were searched up to 1st November 2022 for all types of studies reporting adjusted associations between PNI and mortality or MACE in CAD patients. A random-effect meta-analysis was conducted for PNI as categorical or continuous variable. Subgroup analysis were conducted for multiple confounders.

**Results:**

Fifteen studies with 22,521 patients were included. Meta-analysis found that low PNI was a significant predictor of mortality in CAD patients as compared to those with high PNI (HR: 1.67 95% CI: 1.39, 2.00 *I*^2^ = 95% *p* < 0.00001). Increasing PNI scores were also associated with lower mortality (HR: 0.94 95% CI: 0.91, 0.97 *I*^2^ = 89% *p* = 0.0003). Meta-analysis demonstrated that patients with low PNI had significantly higher incidence of MACE (HR: 1.57 95% CI: 1.08, 2.28 *I*^2^ = 94% *p* = 0.02) and increasing PNI was associated with lower incidence of MACE (HR: 0.84 95% CI: 0.72, 0.92 *I*^2^ = 97% *p* = 0.0007). Subgroup analyses showed mixed results.

**Conclusion:**

Malnutrition assessed by PNI can independently predict mortality and MACE in CAD patients. Variable PNI cut-offs and high inter-study heterogeneity are major limitations while interpreting the results. Further research focusing on specific groups of CAD and taking into account different cut-offs of PNI are needed to provide better evidence.

**Systematic Review Registration:**

No CRD42022365913 https://www.crd.york.ac.uk/prospero/.

## Introduction

Coronary artery disease (CAD) is an extremely common heart ailment affecting millions of individuals around the globe. Estimates indicate that CAD represents 32.7% of all global heart diseases and 1.7% of the overall worldwide disease burden (1). Indeed, CAD is one amongst the most common causes of morbidity and mortality in the elderly and also contributes to a substantial medical and economic burden on the society (2). The disease presents with a varied spectrum depending upon the degree of coronary artery stenosis ranging from stable angina to acute coronary syndrome consisting of unstable angina, non-ST segment elevation myocardial infarction (NSTEMI), or ST segment elevation myocardial infarction (STEMI) (3). The onset of CAD, irrespective of the presentation, has been primarily attributed to poor lifestyle habits leading to oxidative stress, altered lipid metabolism, and thrombosis (4). Recent research also points out to the role of immunity and inflammation in the progression of CAD (5).

Percutaneous coronary intervention (PCI) and coronary artery bypass grafting (CABG) along with medical therapy are the cornerstone of management of CAD. However, despite therapeutic advances, several individuals are at risk for mortality and major adverse cardiac events (MACE) even after early therapy. Early risk stratification which can aid in tailor-made interventions for high-risk patients are needed to improve the prognosis of such individuals. Recently, malnutrition has been shown to be associated with poor prognosis in patients with cardiovascular diseases (6). Hirose et al. (7) has shown that more than 40% of heart failure patients are malnourished and poor nutrition could independently predict mortality. Another study has found that >50% of CAD patients had varying degrees of malnutrition which was predictive of adverse outcomes (8).

Quantification of nutritional status has been a challenge and several tools have been described in literature to classify patients as malnourished (9). One such tool is the prognostic nutritional index (PNI) which allows quantification of the nutritional and immune status of the patient and is calculated by adding the serum albumin and total lymphocyte counts (10). PNI was initially described to check the nutritional status of gastrointestinal surgery, but over the years it has been recognized as an important prognostic factor in several cancers (11–13). Over the past years, many studies have also used PNI to predict prognosis of CAD patients but with inconsistent results (14–18). However, no systematic review has been attempted to summarize the available evidence. Considering this deficiency in literature, this systematic review was conducted to pool data on the ability of PNI to predict mortality and MACE in patients with CAD.

## Materials and methods

### Literature search

The protocol was registered on PROSPERO database (No. CRD42022365913). A comprehensive literature search was undertaken on the webpages of PubMed, Web of Science, Scopus, and Embase for studies to be included in the review. Additionally, Open gray and Google Scholar were checked for gray literature. All searches were from the individual database inception dates up to 1st November 2022. Two reviewers conducted the entire search. There were no language restrictions during the search. Search terms used were “myocardial infarction,” “STEMI,” “NSTEMI,” “coronary artery disease,” “acute coronary syndrome,” “percutaneous coronary intervention,” “PCI,” “coronary artery bypass grafting,” “CABG,” and “prognostic nutritional index.” These were combined by Boolean operators “OR” and “AND” to generate different search strings (Supplementary Table 1). All results were combined electronically and duplicates removed. Initial screening was by titles and abstracts of the studies. Full texts of relevant studies were then cross-examined against the inclusion criteria. All differences were resolved by discussion.

### Inclusion criteria

For inclusion, all study types were eligible. Studies had to report the relationship between PNI and mortality rates or MACE of CAD patients using multivariate adjusted ratios. There was no limitation on the sample size or the treatment of CAD in the included studies. We excluded studies (1) not reporting adjusted ratios (2) not reporting cut-off of PNI if used as a categorical variable (3) not reporting data on either mortality or MACE (4) studies with duplicate data. If there were studies from the same location conducted during the same period the study with the best possible data was to be included.

### Data management

Data noted from the studies was: Details of study authors, database, study location, study type, included patients, treatment provided, sample size, age, gender, comorbidities, patients with myocardial infarction (MI), the cut-off for PNI, definition of MACE, percentage malnourished based on cut-off, follow-up and outcomes.

The Newcastle-Ottawa scale (NOS) was utilized for checking the risk of bias (19). Each article was judged selection of study population, comparability of groups, and outcomes. These were given awarded stars based on predetermined questions with a maximum of four, two and three for each domain, respectively.

### Statistical analysis

The relationship between PNI and mortality or MACE were reported as multivariable-adjusted hazard ratios (HR) by most studies. PNI was used either as a continuous variable or as a categorical variable. A separate meta-analysis for both mortality and MACE was carried out depending upon the type of PNI data (categorical or continuous). Data were pooled to generate combined HR with 95% confidence intervals (CI) in a random-effects model. I^2^ statistic was the tool to check for inter-study heterogeneity. *I*^2^ = 25–50% equaled low, 50–75% equaled medium, and more than 75% denoted substantial heterogeneity. Funnel plots were generated for publication bias analysis. A sensitivity analysis was undertaken to check individual study effects. Sub-group analysis was conducted depending upon study location (Asia and Europe), sample size (>1,000 and < 1,000), diagnosis (MI only and mixed population), treatment given (PCI only and mixed), and PNI cut-off (38, 44–47.1 and 50–51). The data analysis was conducted using “Review Manager” (RevMan, version 5.3; Nordic Cochrane Centre [Cochrane Collaboration], Copenhagen, Denmark; 2014). The review was reported according to the PRISMA recommendations (20).

## Results

### Search details

**Figure 1:**
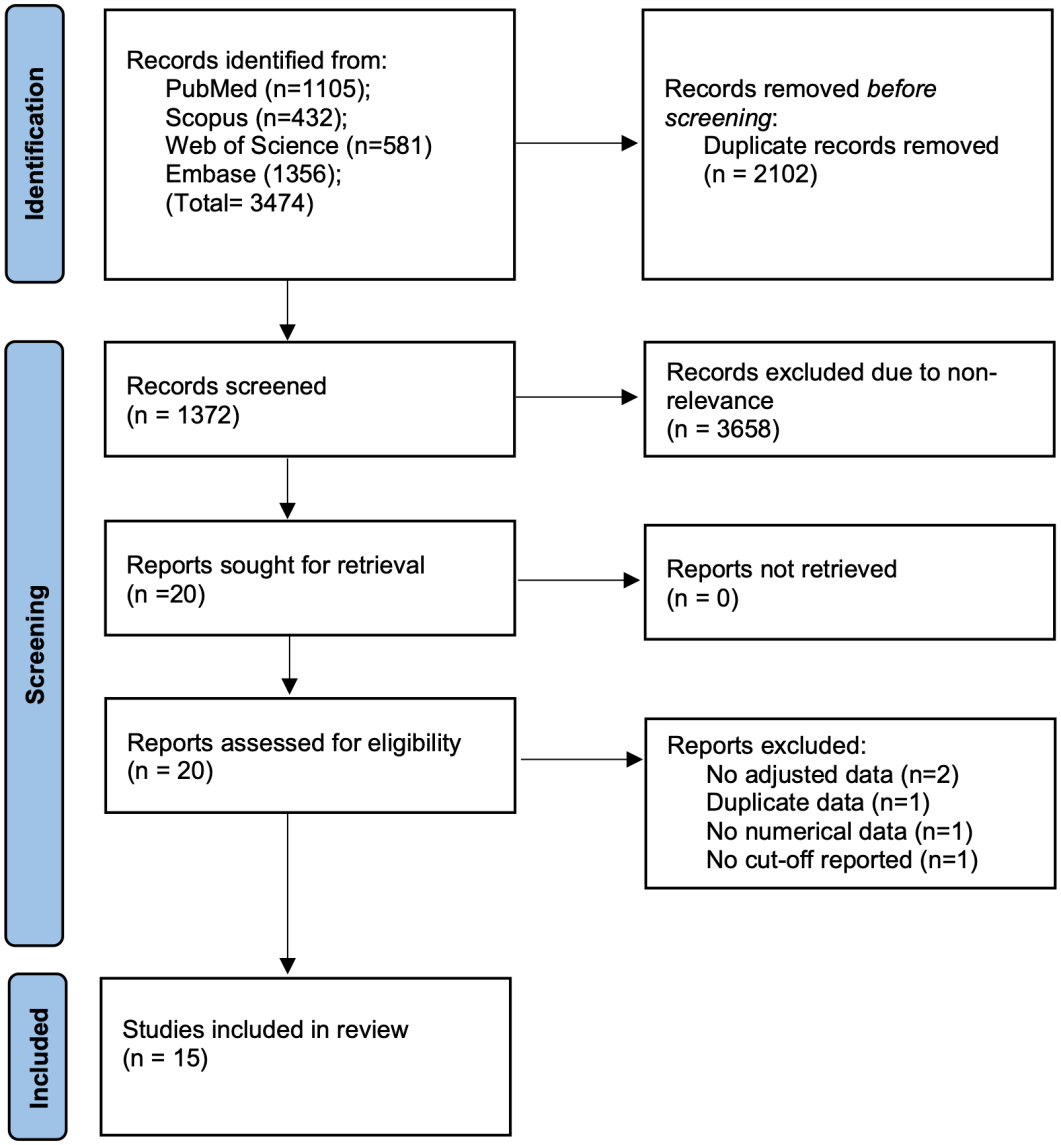
Study flowchart.

3,474 studies search results were obtained by using the mentioned search strategy. Of these, duplicates were removed and 1,372 articles underwent initial screening by the titles and abstracts (Figure 1). Twenty studies were eligible for full-text analysis. Finally, 15 were eligible for this review (14–18, 21–30).

### Study details

All were retrospective observational studies with data restricted from specific countries namely, China, Korea, Japan, Turkey, Spain and Italy (Table 1). The inclusion criteria of the studies included either acute coronary syndrome (ACS), mix of ACS and chronic coronary syndrome (CCS), or MI. Majority studies included patients undergoing PCI while two studies (16, 26) included only CABG patients. In one study (29) the patients were managed by either PCI or CABG while in another (30) PCI or medical management was undertaken. The number of patients in the studies were from 253 to 5,062. The cumulative sample size of the included studies was 22,521 patients. The mean age of included patients was >58 years. There was a predominance of male gender in all studies. The percentage of diabetics in the studies ranged from 19 to 54.9% while the number of hypertensives ranged from 46.7 to 81.9%. Eight of the 15 studies included patients reporting with MI. The cut-off used for PNI was variable ranging from 38 to 50.7. Where follow-up data was reported, the mean or median follow-up was more than 1 year. The definition of MACE was variable in the included studies but included some common variables like death, reinfarction, and stroke (Supplementary Table 2). Majority studies scored a score of 8 on NOS while a few scored 7.

**Table 1:** Details of included studies.

Study	Database	Country	Patient population	Treatment	Sample size	Age (Years)	Male gender (%)	DM (%)	HT (%)	MI (%)	Cut-off of PNI	Mean PNI	Mal-nourished (%)	Follow-up (months)	NOS score
Liu et al. (14)	First Affiliated Hospital of Zhengzhou University	China	ACS, CCS	PCI	3,519	63	68.9	23.5	55.4	100	47.1	NR	33.3	Mean 37.6	8
Kwon et al. (16)	Samsung Medical Center	Korea	ACS, CCS	CABG	2,149	64	77.8	44.8	79.3	11.6	NR	NR	NR	NR	7
Kang et al. (15)	Bundang Medical Center	Korea	ACS	PCI	1930	63	67.9	30.7	58.1	47.5	38	NR	3.9	Median 67.2	8
Chen et al. (18)	The Third People’s Hospital of Chengdu	China	ACS	PCI	799	66.3	72.3	33.8	63.2	NR	38	47.5	4.9	Median 30	8
Tasbulak et al. (26)	Mehmet Akif Ersoy Thoracic and Cardiovascular Surgery Training and Research Hospital	Turkey	ACS, CCS	CABG	586	59.5	78	54.9	77.6	NR	NR	NR	NR	Mean 38	8
Yildirim et al. (17)	Adana Numune Training and Research Hospital	Turkey	NSTEMI	PCI	915	73.1	51.6	32.4	51.9	100	50.7	NR	NR	Mean 64.5	8
Kalyoncuoğlu et al. (28)	Haseki Training and Research Hospital	Turkey	NSTEMI	PCI	253	68.5	71.5	29.2	53.8	100	38	46.9	6.3	Mean 20.5	8
Kim et al. (27)	Gyeongsang National University School of Medicine	Korea	MI	PCI	1,147	65.6	72.5	28.9	46.7	100	50	54	33.3	Median 12	7
Inci et al. (29)	Diskapi Yildirim Beyazid Education and Research Hospital	Turkey	NSTE-ACS	PCI or CABG	498	62.6	69.3	38.1	56.4	77.3	38	NR	44.6	NR	7
Raposeiras Roubín et al. (30)	University Hospital of Vigo	Spain	ACS	PCI or medical management	5,062	66.2	74.5	26.5	64.6	89.4	38	NR	8.9	Median 43	8
Cheng et al. (21)	West China Hospital	China	MI	PCI	598	64	76.4	NR	NR	100	45	47.8	30.4	Median 14.8	8
Wada et al. (22)	Juntendo University Hospital	Japan	CCS	PCI	1988	66.4	82.9	47.9	73.2	0	46.7	48.9*	33.5	Median 90	8
Chen et al. (24)	First Affiliated Hospital of Xinjiang Medical University	China	STEMI	PCI	309	59	80.9	24.9	49.5	100	45	NR	38.8	Median 19.5	8
Keskin et al. (23)	Dr. Siyami Ersek Cardiovascular and Thoracic Surgery Training and Research Hospital	Turkey	STEMI	PCI	1823	58	81	23.6	81.9	100	44	NR	20.9	Median 32.9	8
Basta et al. (25)	Cardiology Department of Massa	Italy	STEMI	PCI	945	65.7	75	19	58	100	38	35.6	NR	Median 24	8

DM, Diabetes mellitus; HT, hypertension; MI, myocardial infarction; NR, not reported; ACS, acute coronary syndrome; CCS, chronic coronary syndrome; STEMI, ST-elevation MI; NSTEMI, non-ST elevation MI; NSTE-ACS, non-ST elevation ACS; PCI, percutaneous coronary intervention; CABG, coronary artery bypass grafting; PNI, prognostic nutritional index; NOS, Newcastle Ottawa scale. *median value.

### Meta-analysis

**Figure 2:**
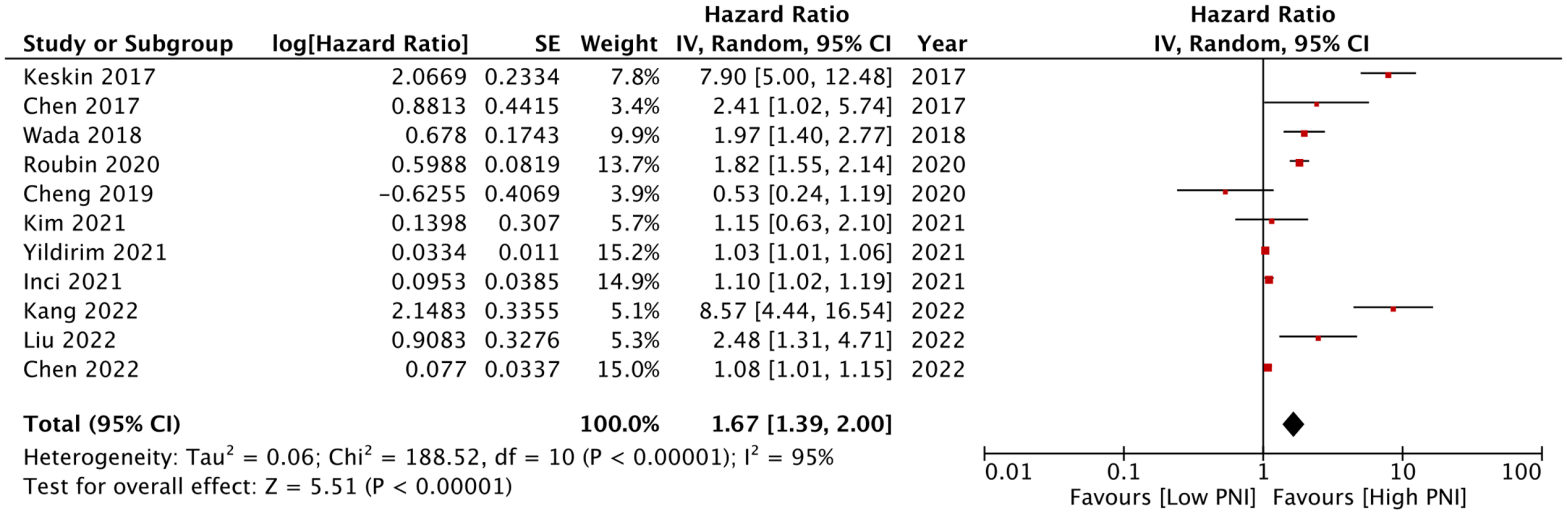
Meta-analysis of mortality with PNI as a categorical variable.

**Figure 3:**
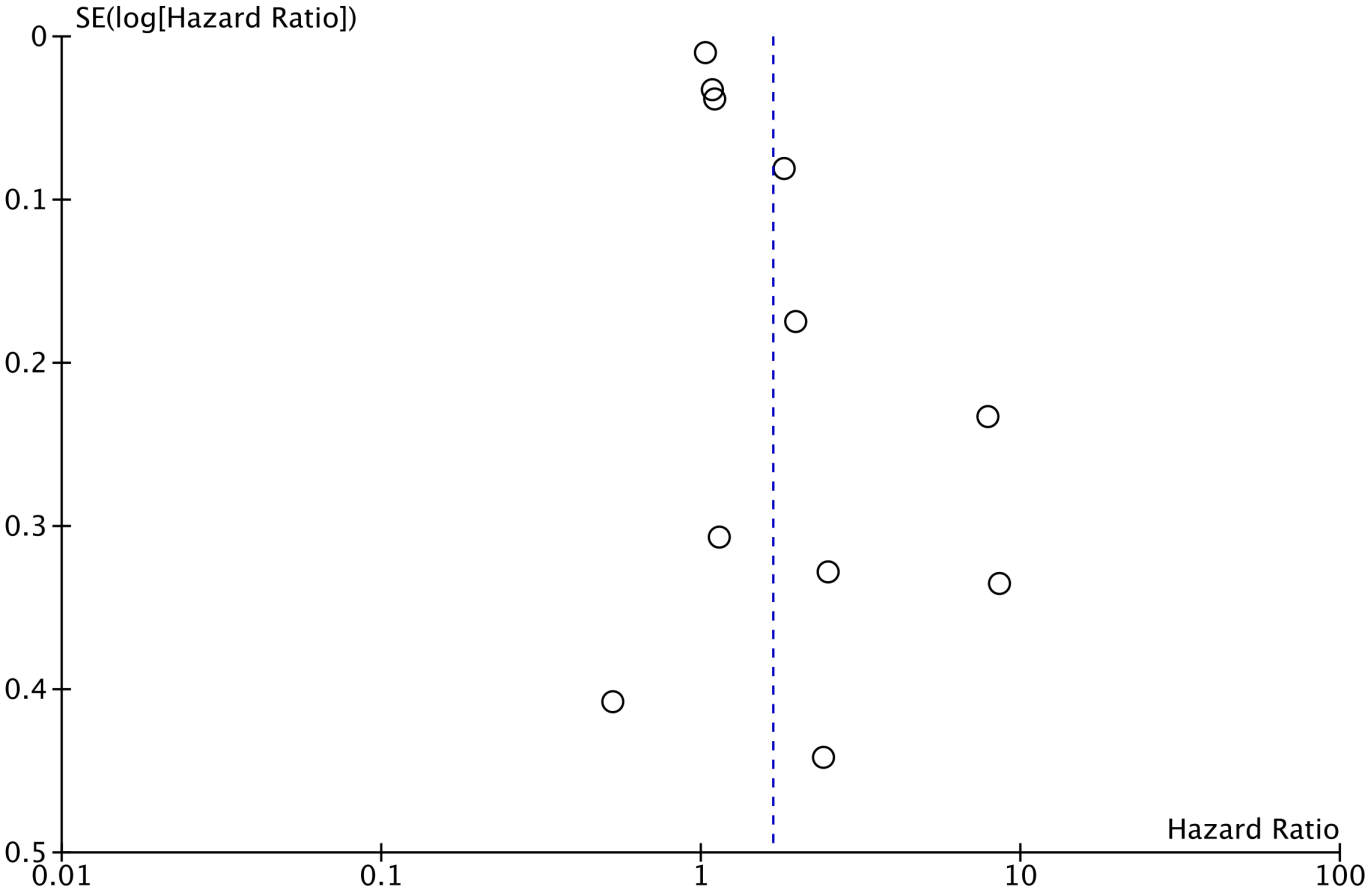
Funnel plot for the meta-analysis of mortality with PNI as a categorical variable.

**Figure 4:**
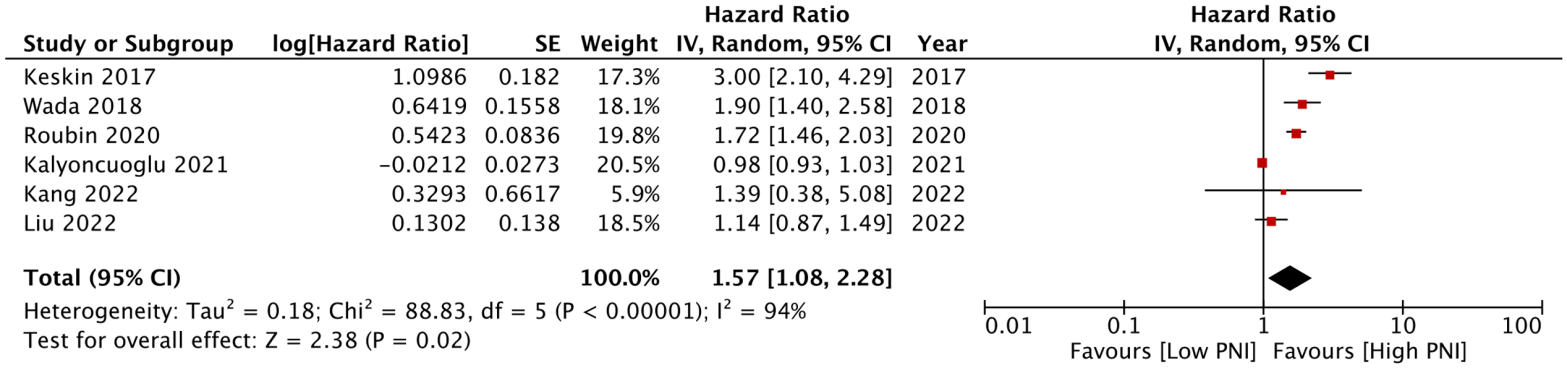
Meta-analysis of MACE with PNI as a categorical variable.

**Figure 5:**
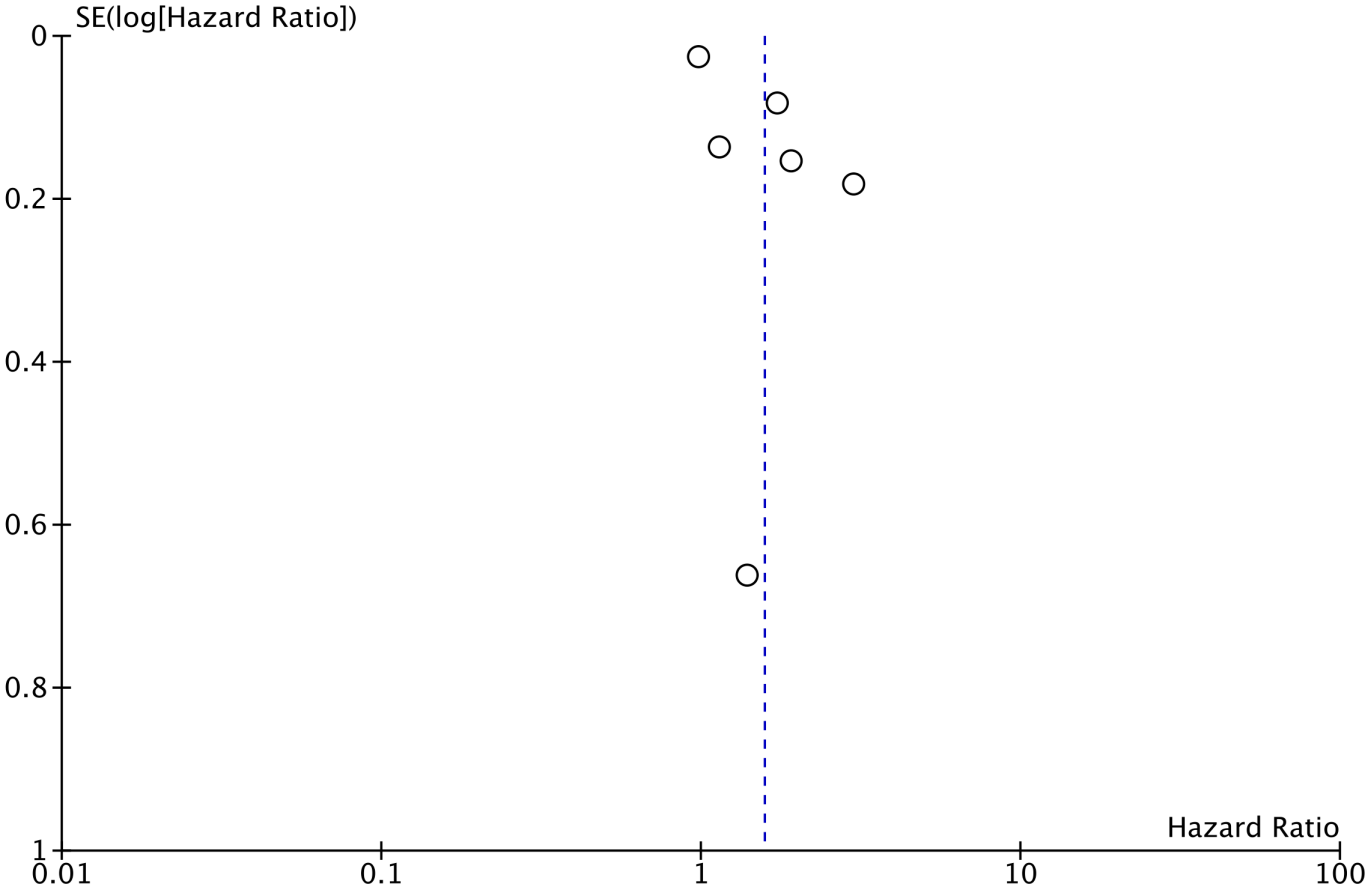
Funnel plot for the meta-analysis of MACE with PNI as a categorical variable.

Eleven studies reported mortality outcome using PNI as a categorical variable and comparing patients with low PNI vs. high PNI. Meta-analysis found that low PNI was associated with significantly higher risk of mortality in CAD patients (HR: 1.67 95% CI: 1.39, 2.00 *I*^2^ = 95% *p* < 0.00001; Figure 2). Funnel plot did not show major asymmetry (Figure 3). Results were the same during sensitivity analysis. Six studies reported MACE outcome with PNI as a categorical variable. Meta-analysis demonstrated significantly higher risk of MACE with low PNI as compared to high PNI (HR: 1.57 95% CI: 1.08, 2.28 *I*^2^ = 94% *p* = 0.02; Figure 4). Funnel plot did not show major asymmetry (Figure 5). The results became non-significant on exclusion of Keskin et al. (23) (HR: 1.37 95% CI: 0.97, 1.94 *I*^2^ = 93% *p* = 0.08) and Roubin et al. (30) (HR: 1.54 95% CI: 0.98, 2.42 *I*^2^ = 93% *p* = 0.06).

**Figure 6:**
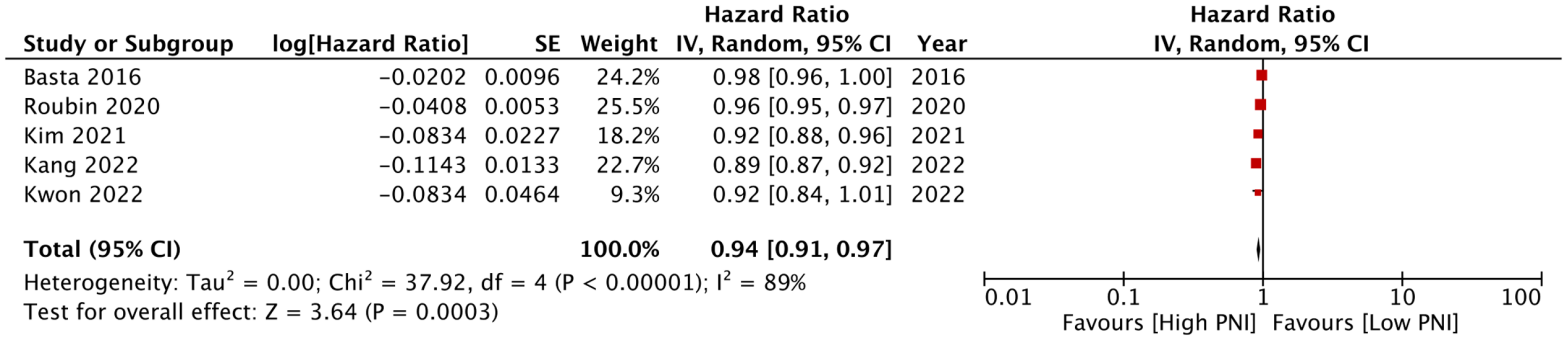
Meta-analysis of mortality with PNI as a continuous variable.

**Figure 7:**
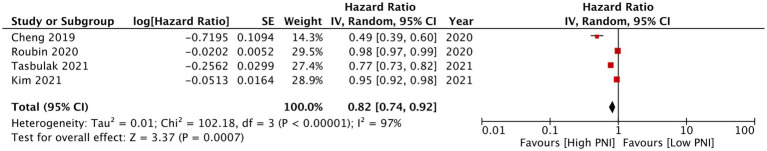
Meta-analysis of MACE with PNI as a continuous variable.

PNI as a continuous variable was used by five studies reporting mortality data. Pooled analysis found that increasing PNI was related with significantly lower mortality rates in CAD patients (HR: 0.94 95% CI: 0.91, 0.97 *I*^2^ = 89% *p* = 0.0003; Figure 6). Similarly, only four studies reported data on MACE with PNI as a continuous variable. Meta-analysis found that increasing PNI was associated with lower incidence of MACE (HR: 0.84 95% CI: 0.72, 0.92 *I*^2^ = 97% *p* = 0.0007; Figure 7). Both results were unchanged on sensitivity analysis.

### Subgroup analyses

All results are demonstrated in Table 2. For mortality data with PNI as a categorical variable, the results were significant on subgroup for Asian and European studies, for studies with sample size >1,000 and < 1,000, and based on diagnosis, i.e., MI only and mixed group. When segregating based on treatment results were significant only for PCI studies but not for studies including other than PCI treatments. Also, when based on PNI cut-off, results were significant for cut-off of 38 and 50–51 but not for 44–47.1.

**Table 2:** Subgroup analysis.

Variable	Groups	Studies	Hazard ratio
Mortality-PNI as categorical variable			
Region	Asia	8	1.78 (95% CI: 1.23, 2.61 *I*^2^ = 92% *p* = 0.02)
	Europe	3	1.62 (95% CI: 1.20, 2.19 *I*^2^ = 97% *p* = 0.002)
Sample size	>1,000	6	2.92 (95% CI: 1.68, 5.09 *I*^2^ = 91% *p* = 0.0001)
	<1,000	5	1.07 (95% CI: 1.00, 1.13 *I*^2^ = 60% *p* = 0.04)
Diagnosis	MI only	5	1.38 (95% CI: 1.08, 1.76 *I*^2^ = 95% *p* = 0.009)
	Mixed	6	2.23 (95% CI: 1.46, 3.40 *I*^2^ = 94% *p* = 0.0002)
Treatment	PCI only	9	1.85 (95% CI: 1.46, 2.35 *I*^2^ = 94% *p* < 0.0001)
	Mixed	2	1.41 (95% CI: 0.86, 2.31 *I*^2^ = 97% *p* = 0.17)
Cut-off	38	4	2.27 (95% CI: 1.33, 3.88 *I*^2^ = 96% *p* = 0.003)
	44–47.1	5	1.88 (95% CI: 0.87, 4.09 *I*^2^ = 95% *p* = 0.11)
	50–51	2	1.03 (95% CI: 1.01, 1.06 *I*^2^ = 0% *p* = 0.02)
Mace-PNI as categorical variable			
Region	Asia	3	1.46 (95% CI: 0.95, 2.23 *I*^2^ = 67% *p* = 0.08)
	Europe	3	1.68 (95% CI: 0.96, 2.93 *I*^2^ = 97% *p* = 0.07)
Sample size	>1,000	5	1.77 (95% CI: 1.30, 2.42 *I*^2^ = 79% *p* = 0.003)
	<1,000	1	0.98 (95% CI: 0.93, 1.03)
Diagnosis	MI only	2	1.69 (95% CI: 0.56, 5.06 *I*^2^ = 97% *p* = 0.35)
	Mixed	4	1.55 (95% CI: 1.20, 2.00 *I*^2^ = 63% *p* = 0.008)
Treatment	PCI only	5	1.54 (95% CI: 0.98, 2.42 *I*^2^ = 93% *p* = 0.06)
	Mixed	1	1.72 (95% CI: 1.46, 2.03)
Cut-off	38	3	1.30 (95% CI: 0.78, 2.17 *I*^2^ = 95% *p* = 0.31)
	44–47.1	3	1.85 (95% CI: 1.07, 3.19 *I*^2^ = 89% *p* = 0.03)
Mortality-PNI as continous variable			
Region	Asia	3	0.90 (95% CI: 0.88, 0.92 *I*^2^ = 0% *p* < 0.0001)
	Europe	2	0.97 (95% CI: 0.95, 0.99 *I*^2^ = 72% *p* = 0.002)
Sample size	>1,000	4	0.92 (95% CI: 0.88, 0.97 *I*^2^ = 90% *p* = 0.001)
	<1,000	1	0.98 (95% CI: 0.96, 1.00)
Diagnosis	MI only	2	0.86 (95% CI: 0.79, 0.94 *I*^2^ = 95% *p* = 0.0003)
	Mixed	3	0.93 (95% CI: 0.87, 0.98 *I*^2^ = 93% *p* = 0.01)
Treatment	PCI only	3	0.93 (95% CI: 0.87, 0.99 *I*^2^ = 94% *p* = 0.03)
	Mixed	2	0.96 (95% CI: 0.95, 0.97 *I*^2^ = 0% *p* < 0.00001)

CI, confidence intervals; MI, myocardial infarction; PCI, percutaneous coronary intervention; PNI, prognostic nutritional index.

Similar meta-analysis of MACE with PNI as a categorical variable revealed insignificant results on subgroup analysis based on region. Results were significant for studies with sample size >1,000, including mixed population of patients, and PNI cut-off of 44–47.1. The remaining results were non-significant (Table 2). For mortality data with PNI as a continuous variable, subgroup analysis showed non-significant results only for smaller sample size study (<1,000). Subgroup analysis for MACE with PNI as a continuous variable was not carried out due to few studies.

## Discussion

The findings of the first meta-analysis assessing the prognostic ability of PNI for CAD patients shows that PNI can be an independently predict mortality and MACE. Despite different cut-offs used by the studies, PNI was a predictor of adverse events when used as a categorical variable. Also, incremental increase in PNI, i.e., better nutritional levels was related with lower incidence of mortality and MACE.

In the past decade, malnutrition has gained widespread attention as a prognostic indicator in multiple diseases (11–13). With a significant proportion of CAD patients being malnourished at the time of treatment (8), it is essential to analyze how nutritional status of the patients impacts mortality and MACE, the two most important outcomes, in individuals with CAD. A wide variety of malnutrition tools have been used in literature for CAD patients and there is no consensus on which is a better tool. Arero et al. (31) recently pooled data from nine studies and found that malnutrition in CAD patients as defined by the Controlling Nutrition Status (CONUT) score could independently predict mortality and MACE. Similarly, Xue et al. (32) in a meta-analysis found sarcopenia to be predictive of adverse outcomes in CAD patients. Another meta-analysis showed the Geriatric Nutritional Risk Index (GNRI) could independently predict mortality and MACE in patients with CAD (33). While CONUT and GNRI are also objective markers of malnutrition like the PNI, these may have limitations in CAD patients. CONUT uses cholesterol levels for assessment of nutritional status and since majority CAD patients are on statin therapy which could alter the cholesterol levels, CONUT may not be an appropriate marker in such patients. GNRI is calculated by combining albumin and body mass index (BMI) values and could possible underestimate malnutrition in individuals with normal or excessively large BMI (22). In this context, PNI could be a readily usable and simple tool to assess malnutrition in CAD patients. Nevertheless, till date, there has been no review assessing the prognostic ability of PNI for such patients.

Following data extraction from the included studies, it was noted that studies used PNI as either a categorical variable with a predefined cut-off to segregate malnourished and normal nutrition patients or used it as a continuous variable to find the association between malnutrition and adverse outcomes. Considering a major difference between the two types of data, separate analyses were carried out to provide a detailed assessment of the association between PNI and outcomes of CAD. In the first part, it was noted that low PNI scores were associated with a 67% increase in mortality and 57% in MACE amongst CAD patients. While when used as a continuous variable, incremental increase in PNI was associated with 9 and 16% decrease in mortality and MACE rates, respectively. These results are in congruence with other malnutrition assessment tools used in CAD patients thereby indicating the PNI can be readily used for risk-stratification of these patients (31, 33). Except for the results of MACE (as categorical variable), all other results maintained their statistical significance on sensitivity analysis. Absence of publication bias also added to the strengths of the review.

Nevertheless, the high inter-study heterogeneity of the meta-analyses cannot be ignored and caution should be exercised while interpreting the results. An endeavor was made to check the source of heterogeneity by conducting various subgroup analyses. For mortality outcomes when PNI was a categorical variable, is was noted that region of the study, sample size, an patient diagnosis did not alter the significance of the results. Studies exclusively on PCI also found PNI to be a significant predictor of mortality. Similar results were noted for the subgroup analysis of mortality with PNI as continuous variable, except for the mixed diagnosis group for which the outcomes turned non-significant but with the upper end of 95% CI very close to 1 (HR: 0.95 95% CI: 0.90, 1.01). Nevertheless, subgroup analysis for MACE (with PNI as categorical variable) showed up multiple non-significant results. This inconsistency could be attributed to the low number of studies in the latter and further reduction in the number of studies on subgroup analyses. Importantly, even when the outcome turned non-significant for different subgroups, the overall effect size was still above 1 indicating a tendency of higher MACE with low PNI.

The most important difference amongst the studies was the variable cut-off of PNI. Studies either used various different cut-offs published in literature or used receiver operating curve analyses to derive an optimal cut-off for their population. An attempt was made to analyze the impact of these cut-offs by segregating them into three close range groups, i.e., 38, 44–47.1, and 50–51. Our analyses revealed non-significant results for mortality (HR: 1.88 95% CI: 0.87, 4.09) and MACE (HR: 1.30 95% CI: 0.78, 2.17) with cut-offs of 44–47.1 and 38, respectively. Such variability in the results are difficult to explain and may be partially attributed to the heterogeneity in the study populations and low number of studies. Since the effect size is >1, it is plausible that further studies generating results with different cut-offs would help strengthen the evidence.

Guidelines for the management of heart failure patients recommend assessment of nutritional status and research also suggest that nutritional intervention strategies may improve prognosis in such patients (34, 35). However, there has been limited research on similar nutritional interventions in CAD patients. Majority research has been directed toward healthy diet and prevention of CAD, but it is unclear at this point how patients with low PNI should be treated. With our research demonstrating poor prognosis of such individuals, there is a need for research on nutritional intervention strategies and its impact on adverse outcomes in CAD patients with low PNI.

This review has notable limitations. First is the high inter-study heterogeneity as discussed earlier. Despite using multiple subgroup analyses, it was not possible to deduce the source of heterogeneity. Baseline differences in study populations, nutritional levels, and treatment protocols may have been the primary reasons. Secondly, the variable cut-offs of PNI was a major limitation. Thirdly, most studies used PCI as the treatment modality. Scarce data was available for CABG and a separate subgroup analysis for the latter could not be performed. Furthermore, we were unable to separate outcomes of ACS and CCS due to inadequate data. This is important since PNI is based on albumin levels and the latter can vary with the diagnosis like in cardiogenic shock. Also important to note is that albumin levels can be affected by congestion during MI and hence the timing of assessment of PNI could be an important variable which needs to be studied. It is also unclear how changes in PNI values during the course of MI affects the results. On the other hand, the second factor of PNI, i.e., lymphocyte levels can also be affected by inflammatory status of atherosclerotic disease or the inflammation caused by ACS. These are important limitations of PNI which need to be considered during clinical practice. Fourthly, there was lack of universal reporting of outcomes in the included studies. Few studies reported only mortality data while other only on MACE. Further variation in the use of PNI as categorical and continuous variable reduced the availability of studies in the meta-analysis thereby decreasing the statistical power. Also, the definition of MACE were not exactly the same amongst included studies with difference in the cause of death included (all-cause, or cardiac only), and inclusion of other variables like heart failure, bleeding and readmission in some studies (14, 27). Such variation could directly affect the results and lead to heterogeneity. Future studies should preferably use similar outcome definitions to generate more accurate results. Lastly, majority data was restricted to few countries in Asian and European region. This can limit the generalization of the results.

Nevertheless, our results have implications for clinical practice. PNI is a simple and easily obtainable prognostic marker which can be obtained in the most basic healthcare setup without a large accompanying cost. This simple marker can provide a rapid early prognostication of the patient predicting both mortality and MACE thereby helping identify those at a greater need for individualized and priority treatment.

## Conclusion

Malnutrition assessed by PNI can independently predict mortality and MACE in CAD patients. Variable PNI cut-offs and high inter-study heterogeneity are major limitations while interpreting the results. Further research focusing on specific groups of CAD and taking into account different cut-offs of PNI are needed to provide better evidence. Studies should also assess if nutritional intervention strategies in patients with low PNI can improve outcomes.

## Data availability statement

The original contributions presented in the study are included in the article/Supplementary material, further inquiries can be directed to the corresponding author.

## Author contributions

SZha and HW conceived and designed the study. SZha, HW, SCh, SCa, CW, and SZho collected the data and performed the analysis. XN was involved in the writing of the manuscript and is responsible for the integrity of the study. All authors have read and approved the final manuscript.

## Conflict of interest

The authors declare that the research was conducted in the absence of any commercial or financial relationships that could be construed as a potential conflict of interest.

## Publisher’s note

All claims expressed in this article are solely those of the authors and do not necessarily represent those of their affiliated organizations, or those of the publisher, the editors and the reviewers. Any product that may be evaluated in this article, or claim that may be made by its manufacturer, is not guaranteed or endorsed by the publisher.
