# Supplementary material for: Prognostic nutritional index and prognosis of patients with coronary artery disease: A systematic review and meta-analysis
Source: Front Nutr. 2023 Mar 16;10:1114053. doi: 10.3389/fnut.2023.1114053 (PMC10061069; doi:10.3389/fnut.2023.1114053)
Supplement: Supplementary file 1 [file Table_1.DOCX]

Supplementary Table 1: Search strategy

| **Search number** | **Query** | **Search Details** |
| --- | --- | --- |
| **1** | ((coronary artery disease) OR (acute coronary syndrome)) AND (prognostic nutritional index) | ("coronary artery disease"[MeSH Terms] OR ("coronary"[All Fields] AND "artery"[All Fields] AND "disease"[All Fields]) OR "coronary artery disease"[All Fields] OR ("acute coronary syndrome"[MeSH Terms] OR ("acute"[All Fields] AND "coronary"[All Fields] AND "syndrome"[All Fields]) OR "acute coronary syndrome"[All Fields])) AND ("nutrition assessment"[MeSH Terms] OR ("nutrition"[All Fields] AND "assessment"[All Fields]) OR "nutrition assessment"[All Fields] OR ("prognostic"[All Fields] AND "nutritional"[All Fields] AND "index"[All Fields]) OR "prognostic nutritional index"[All Fields]) |
| **2** | (((myocardial infarction) OR (STEMI)) OR (NSTEMI)) AND (prognostic nutritional index) | ("myocardial infarction"[MeSH Terms] OR ("myocardial"[All Fields] AND "infarction"[All Fields]) OR "myocardial infarction"[All Fields] OR ("st elevation myocardial infarction"[MeSH Terms] OR ("st"[All Fields] AND "elevation"[All Fields] AND "myocardial"[All Fields] AND "infarction"[All Fields]) OR "st elevation myocardial infarction"[All Fields] OR "stemi"[All Fields] OR "stemis"[All Fields]) OR ("non st elevated myocardial infarction"[MeSH Terms] OR ("non st"[All Fields] AND "elevated"[All Fields] AND "myocardial"[All Fields] AND "infarction"[All Fields]) OR "non st elevated myocardial infarction"[All Fields] OR "nstemi"[All Fields] OR "nstemis"[All Fields])) AND ("nutrition assessment"[MeSH Terms] OR ("nutrition"[All Fields] AND "assessment"[All Fields]) OR "nutrition assessment"[All Fields] OR ("prognostic"[All Fields] AND "nutritional"[All Fields] AND "index"[All Fields]) OR "prognostic nutritional index"[All Fields]) |
| **3** | ((((percutaneous coronary intervention) OR (PCI)) OR (coronary artery bypass grafting)) OR (CABG)) AND (prognostic nutritional index) | ("percutaneous coronary intervention"[MeSH Terms] OR ("percutaneous"[All Fields] AND "coronary"[All Fields] AND "intervention"[All Fields]) OR "percutaneous coronary intervention"[All Fields] OR "PCI"[All Fields] OR ("coronary artery bypass"[MeSH Terms] OR ("coronary"[All Fields] AND "artery"[All Fields] AND "bypass"[All Fields]) OR "coronary artery bypass"[All Fields] OR ("coronary"[All Fields] AND "artery"[All Fields] AND "bypass"[All Fields] AND "grafting"[All Fields]) OR "coronary artery bypass grafting"[All Fields]) OR "CABG"[All Fields]) AND ("nutrition assessment"[MeSH Terms] OR ("nutrition"[All Fields] AND "assessment"[All Fields]) OR "nutrition assessment"[All Fields] OR ("prognostic"[All Fields] AND "nutritional"[All Fields] AND "index"[All Fields]) OR "prognostic nutritional index"[All Fields]) |
