# Supplementary material for: Prognostic nutritional index and prognosis of patients with coronary artery disease: A systematic review and meta-analysis
Source: Front Nutr. 2023 Mar 16;10:1114053. doi: 10.3389/fnut.2023.1114053 (PMC10061069; doi:10.3389/fnut.2023.1114053)
Supplement: Supplementary file 2 [file Table_2.DOCX]

Supplementary Table 2: Definition of MACE in included studies

| Study | Definition of MACE |
| --- | --- |
| Liu 2022(14) | Cardiac death, heart failure, bleeding events, stroke and readmission |
| Kang 2022(15) | Cardiovascular death, myocardial infarction, stroke, and repeat revascularization |
| Tasbulak 2021(26) | All-cause death, myocardial infarction, stroke |
| Kalyoncuoglu 2021(28) | All-cause death, any myocardial infarction, any revascularization and stroke, |
| Kim 2021(27) | Cardiac death, reinfarction, revascularization via repeat percutaneous coronary intervention, heart failure, and cerebrovascular accident |
| Roubin 2020(30) | Cardiovascular mortality, reinfarction, or stroke |
| Wada 2018(22) | All-cause death, myocardial infarction |

MACE, Major adverse cardiac events
